# Growth rate in the dynamical dark energy models

**DOI:** 10.1140/epjc/s10052-014-3127-5

**Published:** 2014-11-05

**Authors:** Olga Avsajanishvili, Natalia A. Arkhipova, Lado Samushia, Tina Kahniashvili

**Affiliations:** 1 Astro Space Center of P.N. Lebedev Physical Institute, Russia, 84/32 Profsoyuznaya str., Moscow, 117997 Russia; 2 Department of Physics, Kansas State University, 116 Cardwell Hall, Manhattan, KS 66506 USA; 3 McWilliams Center for Cosmology and Department of Physics, Carnegie Mellon University, 5000 Forbes Ave, Pittsburgh, PA 15213 USA; 4 Department of Physics, Laurentian University, Ramsey Lake Road, Sudbury, ON P3E 2C Canada; 5 Abastumani Astrophysical Observatory, Ilia State University, 3-5 Cholokashvili Ave., Tbilisi, 0194 Georgia

## Abstract

Dark energy models with a slowly rolling cosmological scalar field provide a popular alternative to the standard, time-independent cosmological constant model. We study the simultaneous evolution of background expansion and growth in the scalar field model with the Ratra–Peebles self-interaction potential. We use recent measurements of the linear growth rate and the baryon acoustic oscillation peak positions to constrain the model parameter $\alpha$ that describes the steepness of the scalar field potential.

## Introduction

Cosmological observations now convincingly show that the expansion of the Universe is accelerating [1–4]. One of the possible explanations of this empirical fact is that the energy density of the Universe is dominated by the so-called *dark energy* (DE) [5, 6], a component with effective negative pressure.

The simplest DE candidate is a time-independent cosmological constant $\Lambda$, and the corresponding cosmological model, the so-called $\Lambda$CDM model, is considered to be a *concordance* model. This simple model, however, suffers from fine tuning and coincidence problems [7, 8]. In the attempt of constructing a more natural model of DE many alternative scenarios have been proposed [9–15].

One of the alternatives to a cosmological constant are the models of a dynamical scalar field. In these models a spatially uniform cosmological scalar field, slowly rolling down its almost flat self-interaction potential, plays the role of a time-dependent cosmological constant. This family of models avoid the fine tuning problem, having a more natural explanation for the observed low energy scale of DE [17, 18, 39–41]. For the scalar field models (the so-called $\phi$CDM model) the equation of state $P_{\phi }= w\rho _{\phi }$ (with $P_\phi$ and $\rho _\phi$ the pressure and energy density of the scalar field) is time dependent, $w= w(t)$, and unlike the cosmological constant, $w (t) \ne -1$, although at late-times it approaches $-1$. When the scalar field energy density starts to dominate the energy budget of the Universe, the Universe expansion starts to accelerate [19, 20]. Even though at low redshifts the predictions of the model are very close to the ones of the cosmological constant, the two models ($\Lambda$CDM and the dynamical DE model) predict different observables over a wide range of redshifts.

The scalar field models can be classified via their effective equation of state parameter. The models with $$ -1<w<-{1}/{3}$$ are referred to as quintessence models, while the models with $$w<-1$$ are referred to as phantom models. The quintessence models can be divided in two broad classes: tracking quintessence, in which the evolution of the scalar field is slow, and thawing quintessence, in which the evolution is fast compared to the Hubble expansion [21–24].

In tracking models the scalar field exhibits tracking solutions in which the energy density of the scalar field scales as the dominant component at the time; therefore the DE is subdominant but closely tracks first the radiation and then matter for most of the cosmic evolution. At some point in the matter domination epoch the scalar field becomes dominant, which results in its effective negative pressure and accelerated expansion [25, 26]. The simplest example of such a model is provided by a scalar field with an inverse-power-law potential energy density $V_{\phi } \propto \phi ^{-\alpha }$, $\alpha >0$ [27], the so-called *Ratra–Peebles* model.

The scalar field models predict a different background expansion history and a growth rate compared to the cosmological constant model ones. Thus the scalar field model can be distinguished from the $\Lambda$CDM model through high precision measurements of distances and growth rates over a wide redshift range [28–38].

In this paper we study generic predictions of slowly rolling scalar field models by taking the Ratra–Peebles model as a representative example. We present a self-consistent and effective way of solving the joint equations for the background expansion and the growth rate. We use a compilation of recent growth rate and baryon acoustic oscillation (BAO) peak measurements to put constraints on the parameter $\alpha$ describing the steepness of the scalar field’s potential.

This paper is organized as follows. In Sect. 2 we investigate in detail the dynamics and the energy of the $\phi$CDM models. In Sect. 3 we study the influence of the $\phi$CDM models on the growth factor of matter density perturbations. In Sect. 4 a comparison is presented of the obtained theoretical results with observational data. We discuss our results and conclude in Sect. 5. We use the natural units with $c= {\hbar }=1$ throughout this paper.

## Background dynamics in $\phi$CDM models

### Background equations

We assume the presence of a self-interacting scalar field $\phi$ minimally coupled to gravity on cosmological scales. The action of this scalar field is given by

1 $$\begin{aligned} S=\frac{M_\mathrm{pl}^2}{16\pi }\int {\mathrm{d}^{4}x\left[ \sqrt{-g}\left( \frac{1}{2}g^{\mu \nu }\partial _\mu \phi \partial _\nu \phi - V(\phi )\right) \right] }, \end{aligned}$$

where $M_\mathrm{pl} =G^{-1/2}$ is the Planck mass, with $G$ being the Newtonian gravitational constant; $V(\phi )$ is the field’s potential. Note that in this presentation the scalar field $\phi$ is dimensionless, and the potential $V(\phi )$ has the $M_\mathrm{pl}^2$ dimension. Following [27] we will assume that the self-interacting potential has a power-law functional form:

2 $$\begin{aligned} V=\frac{\kappa }{2}M_\mathrm{pl}^2\phi ^{-\alpha }, \end{aligned}$$

where $\alpha >0$ is a model parameter that determines the steepness of the scalar field potential. Compliance with current observational data requires $\alpha \le 0.7$ [39–41]. The larger value of $\alpha$ induces the stronger time dependence of the equation of state parameter $w_{\phi }$, while $\alpha$=0 corresponds to the $\Lambda$CDM case. Another model parameter $\kappa >0$ is a positive dimensionless constant which is related to $\alpha$ (see the appendix and Ref. [42] for its dependence on $\alpha$).

We assume the flat and isotropic Universe that is described by the standard Friedmann–Lemaître–Robertson–Walker homogeneous cosmological spacetime model (FLRW) $\mathrm{d}s^2=-\mathrm{d}t^2+a(t)^2\mathrm{d}\mathbf{x}^2$, and we normalize the scale factor to be equal to 1 at present time, $a_\mathrm{today}=a_0=1$, i.e. $a=1/(1+z)$, where *z* is the redshift.

Using the action for the scalar field, Eq. (1), we obtain the Klein–Gordon equation (equation of motion) for the scalar field

3 $$\begin{aligned} \ddot{\phi }+3H{\dot{\phi }}+\frac{\partial V(\phi )}{\partial \phi }=0, \end{aligned}$$

where an over-dot represents the derivative with the respect of physical time $t$; $H(a)=H_0 E(a)= {\dot{a}}/a$ is the Hubble parameter and $H_0$ is its value today.

The flatness of the Universe requires that the total energy density of the Universe is equal to the critical energy density, i.e. $\rho _\mathrm{tot}$ = $\rho _\mathrm{cr}$ = $3H_0^2 M_\mathrm{pl}^2/ (8\pi )$. We also introduce the energy density parameters for each component as $\Omega _i = \rho _i/\rho _\mathrm{cr}$ (where the index $i$ denotes the individual components, such as radiation, matter or the scalar field).

The energy density and pressure of the scalar field are given by

4 $$\begin{aligned} \rho _\phi&= \frac{M_\mathrm{pl}^2}{32\pi } \left( \dot{\phi }^2/2 + V(\phi ) \right) , \end{aligned}$$

5 $$\begin{aligned} P_\phi&= \frac{M_\mathrm{pl}^2}{32\pi } \left( \dot{\phi }^2/2 - V(\phi ) \right) . \end{aligned}$$

The corresponding equation of state is given by $w=({\dot{\phi }}^2/2 - V(\phi ))/({\dot{\phi }}^2/2 + V(\phi )).$ It is clear that the requirement $w_\mathrm{today} \simeq -1$ imposes the constraint ${\dot{\phi }}^2/2 \ll V(\phi )$.

The first Friedmann equation implies

6 $$\begin{aligned} E^2(a)= \Omega _{r0} a^{-4} + \Omega _{m0} a^{-3} + \Omega _\nu (a) + \Omega _\phi (a), \end{aligned}$$

where $\Omega _{r0}$ and $\Omega _{m0}$ are the radiation and matter (including all non-relativistic components, except neutrinos, which were relativistic at the early stages) density parameters today, while $\Omega _\nu$ is the total neutrino energy density which scales as $\propto a^{-4}$ before neutrinos becoming non-relativistic, and thereafter evolves as $a^{-3}$. The scalar field energy density parameter is given by

7 $$\begin{aligned} \Omega _\mathrm{\phi }(a) =\frac{1}{12H_0^2}\left( \dot{\phi }^2+\kappa M_\mathrm{pl}^2\phi ^{-\alpha }\right) . \end{aligned}$$

To ensure the flatness of the Universe, we require that $\Omega _{m0} + \Omega _{\nu 0} = 1- \Omega _{\phi 0}$, where $\Omega _{\nu 0}$ and $\Omega _{\phi 0}$ are the current energy density parameters for neutrinos and the scalar field, respectively. Since in the standard cosmological scenario the neutrino density is believed to be negligible compared to the matter and DE densities at low redshifts, we will ignore this component in our computations from now on (as well we neglect the radiation contribution to today’s energy density).

#### Initial conditions

We integrate the set of equations Eqs. (3) and (6) numerically, starting from a very early moment $a_\mathrm{in}=5 \times 10^{-5}$ to the present time $a_0=1$. For the scalar field we assume the following initial conditions:

8 $$\begin{aligned} \phi _\mathrm{in}&= \left[ \frac{1}{2}\alpha (\alpha +2)\right] ^{1/2}a_\mathrm{in}^{\frac{4}{\alpha +2}}, \end{aligned}$$

9 $$\begin{aligned} { \phi }_\mathrm{in}^\prime&= \left( \frac{2\alpha }{\alpha +2}\right) ^{1/2}a_\mathrm{in}^{\frac{2-\alpha }{2+\alpha }}, \end{aligned}$$

where a prime denotes differentiation with respect to the scale factor a. We also used $a(t) \propto t^{1/2}$ as consistent with a radiation dominated epoch. These initial conditions were derived from Eq. (3) (for details see Appendix A). We fix the values of the parameters, $\Omega _{m0}=0.315$, $\Omega _{\phi 0}=0.685$, $h=0.673$, to the best-fit values obtained by Planck collaboration [43].

#### The results of computations of the dynamics and the energy of the $\phi$CDM model.

**Fig. 1:**
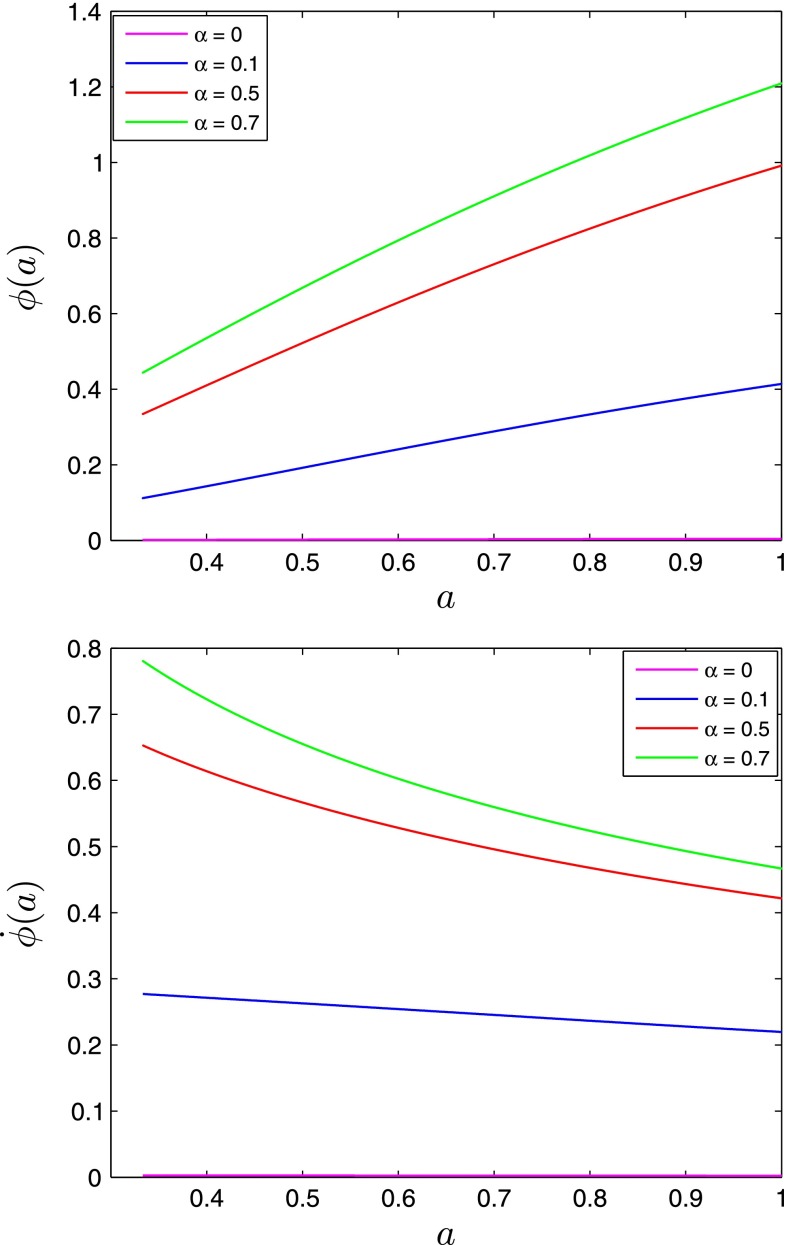
The scalar field amplitude $\phi (a)$ (*top panel*) and its time-derivative $\dot{\phi }(a)$ (*bottom panel*) for different values of the $\alpha$ parameter

**Fig. 2:**
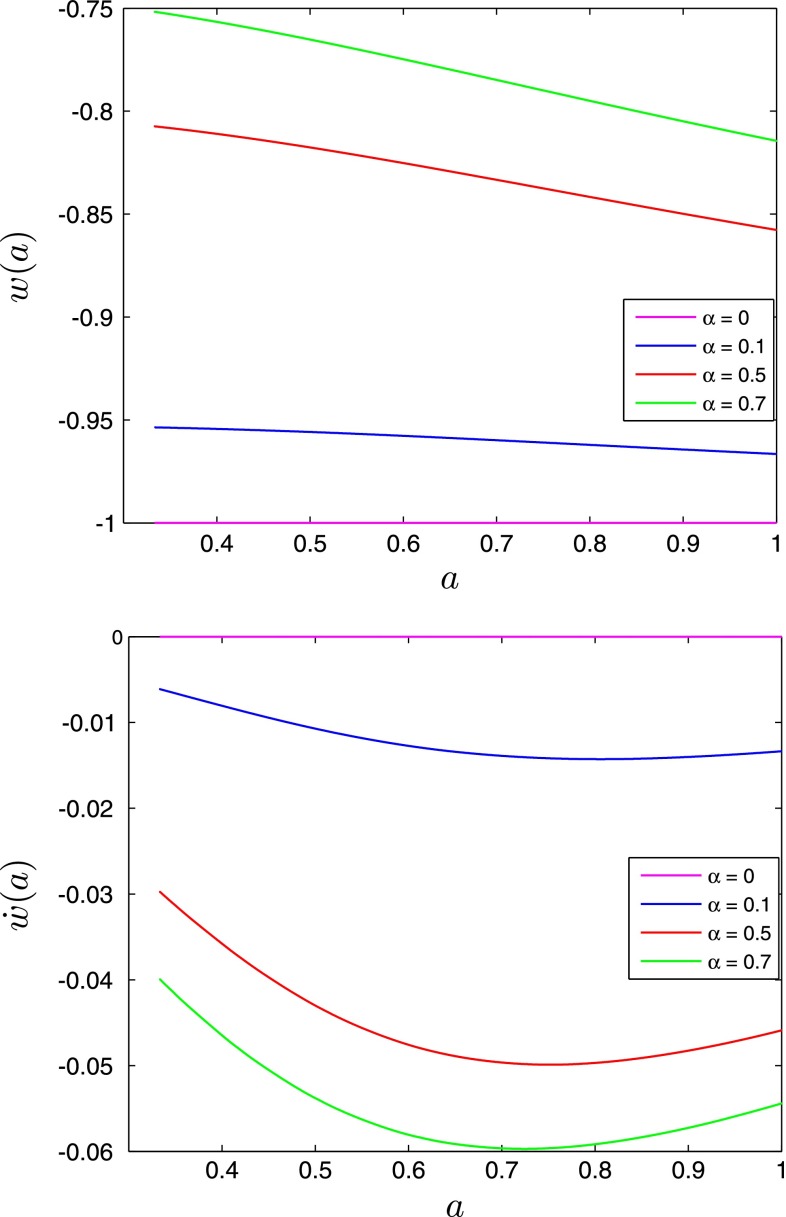
DE equation of state parameter $w(a)$ (*top panel*) and its time-derivative $\dot{w}(a)$ (*bottom panel*) as a function of scale factor for different values of the $\alpha$ parameter

**Fig. 3:**
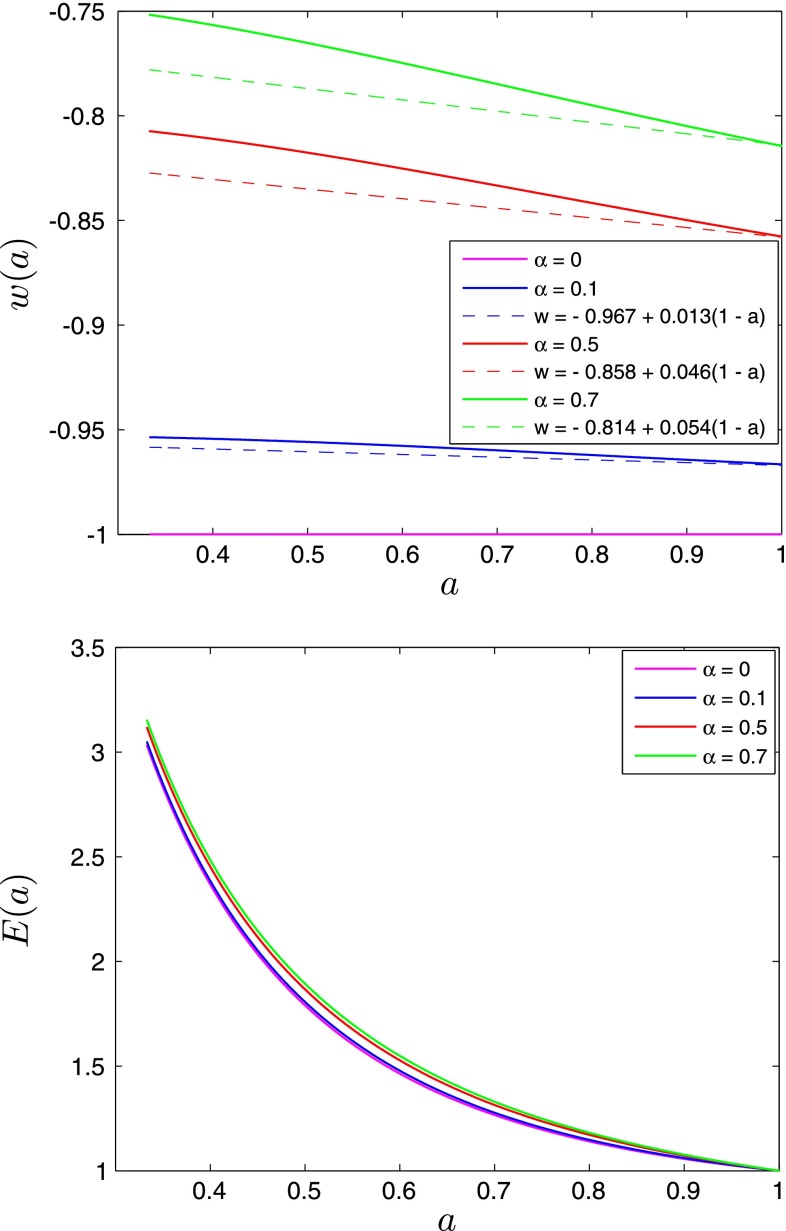
On the *top panel* is shown $w(a)$ for different values of $\alpha$ parameter along with predictions computed from the CLP parametrization with corresponding best-fit values for $w_0$ and $w_{a}$. On the *bottom panel* is shown the normalized Hubble expansion rate $E(a)$ for different model parameters $\alpha$

**Fig. 4:**
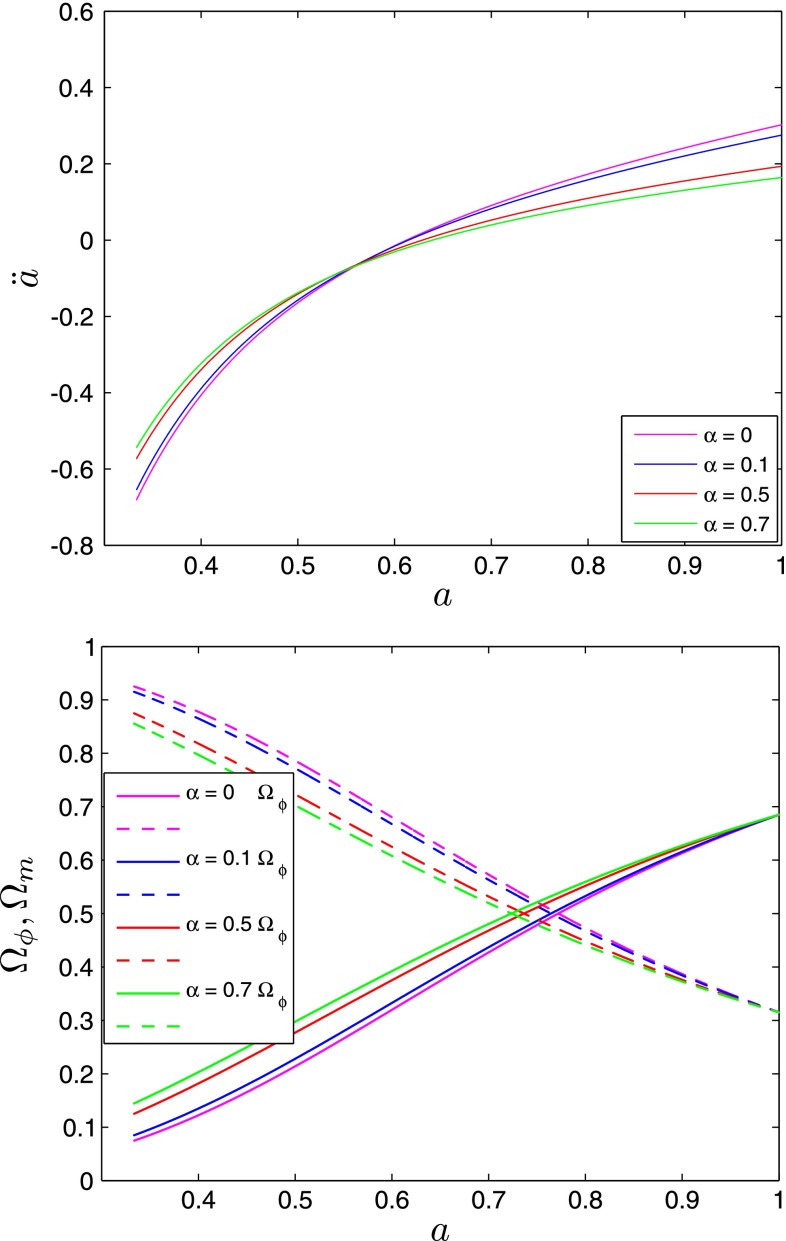
The second derivative of the scale factor (*top panel*) and energy densities of $\Omega _m(a)$ (*dashed lines*) matter and $\Omega _\mathrm{\phi }(a)$ (*solid lines*) scalar field (*bottom panel*) as functions of scale factor for different values of the $\alpha$ parameter

We present the background dynamics in the presence of the scalar field DE on Figs. 1, 2, 3, and 4. The large values of the $\alpha$ parameter imply larger values of the scalar field amplitude $\phi (t)$ and its time derivative $\dot{\phi }(t)$ at all redshifts. The large values of the $\alpha$ parameter result also in the large values of $w$ and $\mathrm{d}w/\mathrm{d}a$ at all redshifts.

The evolution of the equation of state $w(a)$ is presented on Fig. 3. We find that for all values of the $\alpha$ parameter, the Chevallier–Polarsky–Linder (CPL) parametrization of the DE equation of state $w(a)=w_0+w_a(1-a)$ near $a=1$ (where $w_0=w(a=1)$ and $w_a=(-\mathrm{d}w/\mathrm{d}a)|_{a=1}$) [44–46] provides a good approximation in the range of the scale factor $a = [0.98$–$1]$.

The evolution of $E(a)$ for different values the $\alpha$ parameters is shown on Fig. 3. As we can expect the expansion of the Universe occurs more rapidly with increasing value of the $\alpha$ parameter, the $\Lambda$CDM limit corresponding to the slowest rate of the expansion. The value of the $\alpha$ parameters affects also the redshift of the equality between matter and scalar field energy densities (see Fig. 4); with larger values of $\alpha$ the scalar field domination begins earlier and vice versa.

## Growth factor of matter density perturbations in dark energy models

We use the linear perturbation equations for matter overdensities [47, 48] to describe the evolution of small overdensities in a homogeneous expanding Universe,

10 $$\begin{aligned} \delta ^{''}+\left( \frac{3}{a}+\frac{E^{'}}{E}\right) \delta ^{'}-\frac{3\Omega _{m,0}}{2a^{5}E^{2}}\delta =0, \end{aligned}$$

where $\delta \equiv \delta \rho _m/\rho _m$, with $\rho _m$ and $\delta \rho _m$ the density and overdensity of the matter component, respectively.

Following [47] we use the initial conditions $\delta (a_\mathrm{in})=\delta ^{'}(a_\mathrm{in})=5\times 10^{-5}$, with $a_\mathrm{in}=5\times 10^{-5}$ as defined above.

We define by $D(a)=\frac{\delta (a)}{\delta (a_i)}$ the linear growth rate, which shows how much the perturbations have grown since initial moment $a_\mathrm{in}$. We normalize the growth rate so that $D(a_\mathrm{in})=1$. The fractional matter density $f_1(a) \equiv \Omega _m(a)$ as a function of time is given by $f_1(a)=\Omega _{m0}a^{-3}/E^2,$ and we define the function $f_2(a)$, which describes the growth rate of the matter perturbations, as a logarithmic derivative of linear growth rate [49]: $f_2(a) ={dlnD(a)}/{dlna}.$ In $\Lambda$CDM cosmology the two functions can are related by

11 $$\begin{aligned} f_2(a) \approx [f_1(a)]^{\gamma }. \end{aligned}$$

The $\gamma$ parameter is also referred to as the growth index [50], and it depends on both the model of DE and the theory of gravity. In general relativity (GR) the time dependence of the $\gamma$ index can be fit by [50]

12 $$\begin{aligned} \gamma =0.55+0.05(1+w_0+0.5w_a), ~~\mathrm{if}~~ w_0 \ge -1. \end{aligned}$$

For the $\Lambda$CDM model (with $w=-1$), the growth index is $\gamma = 0.55$ [50, 51]. The $\phi$CDM model has been tested through the growth rate in Ref. [52]. In more complex coupled dark energy models, the growth rate has been studied in Refs. [53–55]. The measured value of $\gamma$ in conjunction with tight constraints on the other cosmological parameter can be used to test the validity of GR; see Refs. [56, 57] for recent studies to use the linear growth rate data to determine the deviation of the theory of gravity on extragalactic scales from the standard GR.

### The results of computations of the growth factor of matter density perturbations in $\phi$CDM dark energy model

**Fig. 5:**
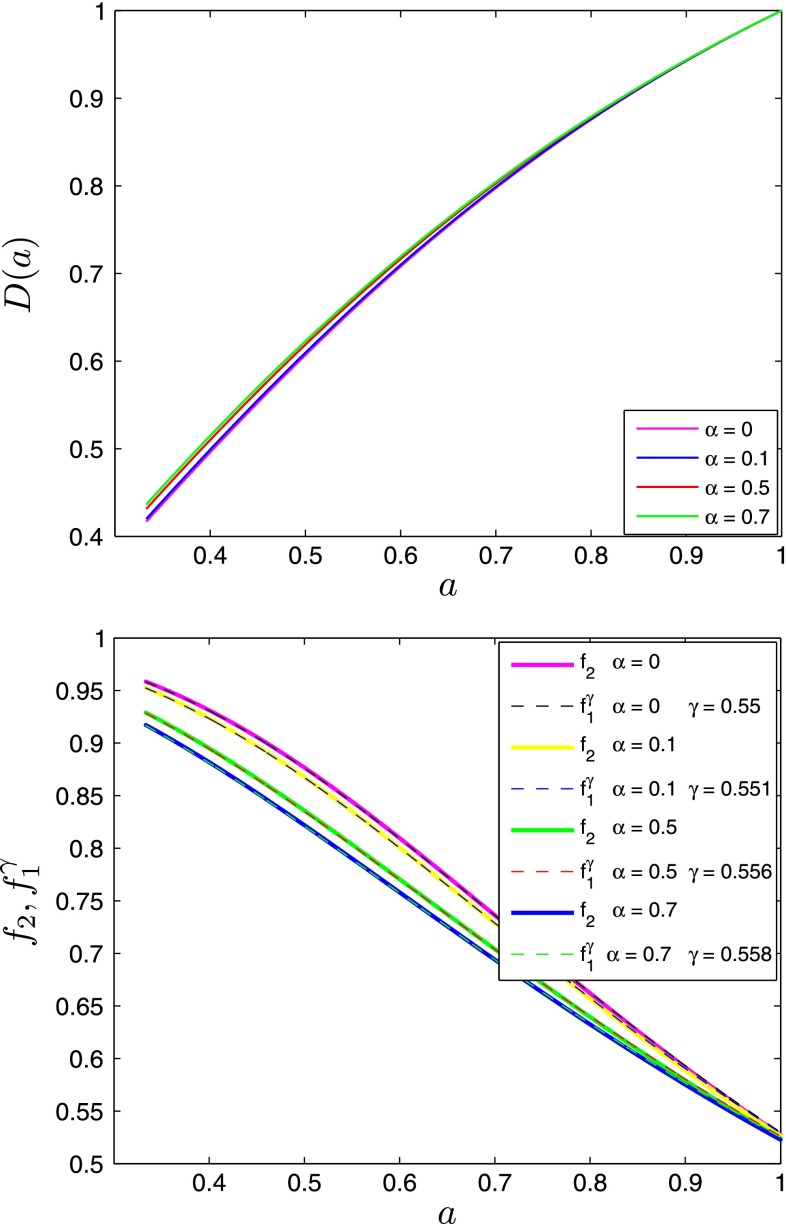
On the *top panel* is shown the linear growth as $D(a)$ as a function of scale factor for different values of the $\alpha$ parameter. On the *bottom panel* is shown the logarithmic growth rate as a function of the scale factor for different values of the $\alpha$ parameter $f_2$ (*solid lines*) along with the predictions $f_1^\gamma$ (*dashed lines*), computed for the corresponding best-fit values of the $\gamma$ parameter

We present the solutions of the growth Eq. (10) in RP models on Fig. 5.

**Fig. 6:**
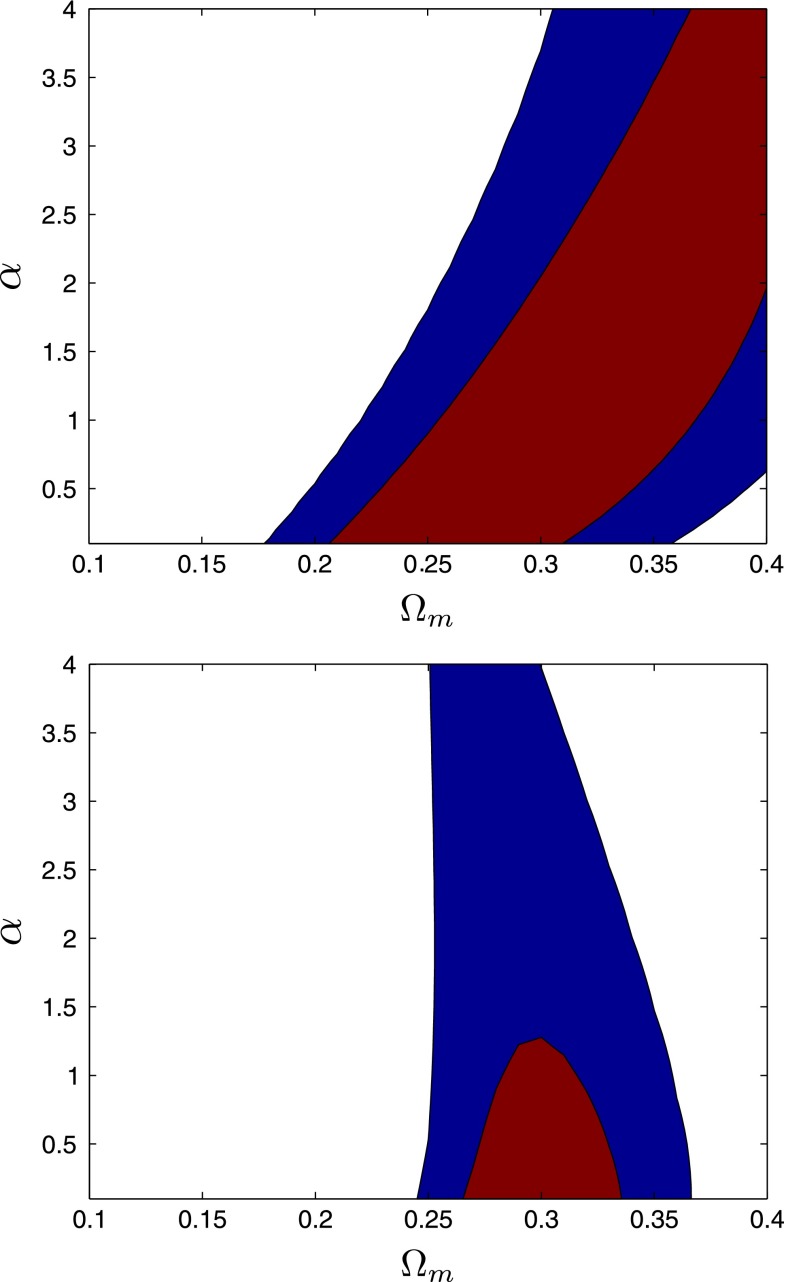
1$\sigma$ and 2$\sigma$ confidence level contours on parameters $\Omega _{m}$ and $\alpha$ of $\phi$CDM model. On the *top panel* are shown the constraints we obtained from the growth rate data [59]. On the *bottom panel* are shown the constraints, obtained after adding BAO measurements and CMB distance prior as in [60] for the BAO/CMB distance prior

We have checked that the power-law approximation Eq. (11) works well for the scalar field DE. The effective value of the growth index $\gamma$ depends on $\alpha$ and is slightly higher than the $\Lambda$CDM limit of 0.55 (Fig. 6).

## Comparison with observations

The $\phi$CDM models generically predict a faster expansion rate and a slower rate of growth at low redshifts. Tight measurements of the expansion rate, distance–redshift relationship and the growth rate at multiple redshift ranges can be used to simultaneously constrain the background dynamics and the growth of structure and discriminate between $\phi$CDM and $\Lambda$CDM models.

For the rest of this section we will concentrate specifically on the discriminative power of the growth rate and BAO measurements from galaxy surveys. For simplicity we will assume that the spatial curvature is known precisely and $\Omega _{k} = 0$. Pavlov et al. [58] explored in detail the background dynamics and the growth of structure of the generalized non-flat $\phi$CDM model. We take a compilation of growth rate measurements from [59] and obtain a posterior likelihood function of the parameters $\alpha$ and $\Omega _{m}$. To do this we apply the same method as [59]; we numerically solve Eq. (10) for series of $\alpha$ and $\Omega _{m}$ values and compute a $\chi ^2$ value,

13 $$\begin{aligned} \chi ^2(\alpha ,\Omega _{m}) = \frac{[f_{m} - f_\mathrm {th}(\alpha ,\Omega _{m})]^2}{\sigma _f^2}, \end{aligned}$$

where $f_{m}$ is the measured value of the growth rate, $f_\mathrm {th}$ a theoretically computed value, and $\sigma _f^2$ one standard deviation error of the measurement. Assuming that the likelihood is Gaussian we have

14 $$\begin{aligned} \mathcal {L}^\mathrm {f}(\alpha ,\Omega _{m})\propto \mathrm {exp}[-\chi ^2(\alpha ,\Omega _{m})/2]. \end{aligned}$$

The 1$\sigma$ and 2$\sigma$ confidence contours resulting from this likelihood are presented on the top panel of Fig. 5. The likelihood contours in the $\alpha$–$\Omega _{m}$ plane obtained from the growth rate data alone are highly degenerate. If we fix $\alpha =0$ we get $\Omega _{m} = 0.278 \pm 0.03$, which is within 1$\sigma$ of the best-fit value obtained by the Planck collaboration [43]. Values of $\Omega _{m} < 0.2$ are ruled out at more than $2\sigma$ confidence level, but large values of $\Omega _{m}$ are still allowed as long as $\alpha$ is large.

To break the degeneracy between the $\Omega _{m}$ and $\alpha$ parameters we now add a compilation of low-redshift BAO measurements from [60]. We follow the same approach as [60]; we compute the angular distance

15 $$\begin{aligned} d_{A}(z, \alpha , \Omega _{m}, H_0) = c\int _0^z \frac{\mathrm{d}z'}{H(z', \alpha , \Omega _{m}, H_0)}, \end{aligned}$$

and a distance scale

16 $$\begin{aligned}&D_{V}(z, \alpha , \Omega _{m}, H_0) \nonumber \\&\quad =[d_{A}^2(z, \alpha , \Omega _{m}, H_0)cz/H(z, \alpha , \Omega _{m}, H_0)]^{1/3}, \end{aligned}$$

at a series of redshifts and construct a combination $\eta (z) \equiv d_{A}(z_\mathrm {bao})/D_{V}(z_\mathrm {bao})$ where $H(z)$ is the Hubble parameter and $H_0$ is a Hubble constant. Assuming Gaussianity of the error bars we again compute the $\chi ^2$,

17 $$\begin{aligned} \chi ^2_\mathrm {bao} = \varvec{X}^\mathrm {T}\varvec{C}^{-1}\varvec{X} \end{aligned}$$

and a likelihood function

18 $$\begin{aligned} \mathcal {L}^\mathrm {bao}(\alpha ,\Omega _{m},H_0) \propto \mathrm {exp}(-\chi ^2_\mathrm {bao}/2), \end{aligned}$$

where $\varvec{X} = \eta _\mathrm {th} - \eta _{m}$ and $\varvec{C}$ is the covariance matrix of the measurements. To marginalize over the parameter $H_0$ in $\mathcal {L}^\mathrm {bao}$ we take a Gaussian prior of $H_0 = 74.3 \pm 2.1$ from [61]. We assume that $\mathcal {L}^\mathrm {f}$ and $\mathcal {L}^\mathrm {bao}$ are independent and the combined likelihood is simply a product of the two. The results are presented on the bottom panel of Fig. 5. The addition of BAO measurements breaks the degeneracy in the growth rate data. $\Omega _{m}$ is now constrained to be within $0.26 < \Omega _{m} < 0.34$ at 1$\sigma$ confidence level. For the $\alpha$ parameter we get $0\le \alpha \le 1.3$ at 1$\sigma$ confidence level.

## Discussion and conclusions

We explored observable predictions of the scalar field DE model. We showed that the model differs from $\Lambda$CDM in a number of ways that are generic and do not depend on the specific values of model parameters. For example, in scalar field models the expansion rate of the Universe is always faster and the DE dominated epoch sets in earlier than in $\Lambda$CDM model when other cosmological parameters are kept fixed. The two models also differ in their predictions for the growth rate, where the scalar field model generically predicts a slower growth rate than $\Lambda$CDM.

We used a compilation of BAO, growth rate, and the distance prior from the CMB to constrain the model parameters of the scalar field model. We find that if only the growth rate data is used there is a strong degeneracy between $\Omega _{m}$ and $\alpha$, where higher values of $\alpha$ are allowed as long as the $\Omega _{m}$ parameter is large as well. When combining these constraints with the constraints coming from a distance–redshift relationship (BAO data and the distance prior from CMB) the degeneracy is broken and we get $\Omega _{m} = 0.30 \pm 0.04$ and $\alpha < 1.30$ with a best-fit value of $\alpha = 0.00$.

## Appendix A: Calculation of $\kappa$ factor

In the appendix we calculate the $\kappa$ factor following Sect. 3.6.3 of Ref. [42]. Let us represent the scale factor and the scalar field $\phi (t)$ in the power-law forms,

19 $$\begin{aligned} a(t)=a_\star \left( \frac{t}{t_\star }\right) ^n,\quad \phi (t)= \phi _\star \left( \frac{t}{t_\star }\right) ^p \end{aligned}$$

where $a_\star \equiv a(t_\star )$ and $\phi _\star \equiv \phi (t_\star )$ are the scale factor and the scalar field values at $t=t_\star$. Equation (3) implies $p=2/(2+\alpha )$ (see for details Sect. 3.6.3 of Ref. [42]), and respectively,

20 $$\begin{aligned} \phi _\star ^{\alpha +2}=\frac{(\alpha +2)^2}{4(6n+3n\alpha -\alpha )}\kappa \alpha M_\mathrm{pl}^2 t_\star ^2. \end{aligned}$$

Using Eq. (19) with Eq. (20) with Eqs. (4) and (6), we obtain

21 $$\begin{aligned}&\rho = \frac{3n}{8\pi }\left( \frac{M_\mathrm{pl}}{t_\star } \right) ^2 \frac{\phi _\star ^2}{\alpha (\alpha +2)} \left( \frac{t}{t_\star }\right) ^{\frac{-2\alpha }{\alpha +2}} \end{aligned}$$

22 $$\begin{aligned}&\left( \frac{n}{t}\right) ^2 = \frac{8\pi }{3M_\mathrm{pl}^2}\rho , \end{aligned}$$

where $\rho \equiv \rho _\phi$ and we assume that it is the energy density of a single component that was dominant at $t<t_\star$ in the Universe. Assuming $\rho (t) = \rho _\star ({t}/{t_\star })^\beta$ we get $\beta = -2\alpha /(\alpha +2)$. On the other hand, assuming that the component energy density is $\rho _\star$ at $a=a_\star$, and accounting for the dominance of the component at this epoch, we have

23 $$\begin{aligned} \rho =\rho _{\star }\left( \frac{a_\star }{a}\right) ^{\frac{2}{n}}, \end{aligned}$$

where $n=1/2$ is for radiation and $n=2/3$ for the matter dominant epochs. Expressing $1/t^2$ through Eq. (22), and using Eq. (23) in Eq. (21) with assuming $a=a_\star$, $\rho =\rho _{\star }$, we can derive $\phi _\star ^2$ and comparing the obtained result with Eq. (20), we find

24 $$\begin{aligned} \kappa =\frac{32 \pi }{3nM_\mathrm{pl}^4} \left( \frac{6n+3n\alpha -\alpha }{\alpha +2}\right) [n\alpha (\alpha +2)]^{\frac{\alpha }{2}} \rho _{\star }. \end{aligned}$$

Plugging Eq. (24) in Eq. (20), and using Eq. (22), we obtain

25 $$\begin{aligned}&\phi _\star = [n\alpha (\alpha +2)]^{\frac{1}{2}},\end{aligned}$$

26 $$\begin{aligned}&\phi =[n\alpha (\alpha +2)]^{\frac{1}{2}} \left( \frac{a}{a_\star }\right) ^{\frac{2}{n(\alpha +2)}} \end{aligned}$$

that simultaneously lead to initial conditions, Eqs. (8) and (9) in the radiation dominated epoch with $n=1/2$ through assuming $a_\star =a_0$.

Plugging Eq. (26) in Eq. (3),

27 $$\begin{aligned} \kappa =\frac{4n}{M_\mathrm{pl}^2 t_\star ^2} \left( \frac{6n+3n\alpha -\alpha }{\alpha +2}\right) [n\alpha (\alpha +2)]^{\alpha /2}. \end{aligned}$$

Since Eq. (24) must be valid for any $t_\star$, we imply the freedom of our choice and use $t_\star =M_\mathrm{pl}^{-1}$. Finally we have for $n=1/2$ and $n=2/3$, respectively:

28 $$\begin{aligned} \kappa (n=1/2)&= \left( \frac{\alpha +6}{\alpha +2}\right) \left[ \frac{1}{2}\alpha (\alpha +2)\right] ^{\alpha /2},\end{aligned}$$

29 $$\begin{aligned} \kappa (n=2/3)&= \frac{8}{3} \left( \frac{\alpha +4}{\alpha +2}\right) \left[ \frac{2}{3}\alpha (\alpha +2)\right] ^{\alpha /2}. \end{aligned}$$
